# Electronic Tongue for Direct Assessment of SARS-CoV-2-Free and Infected Human Saliva—A Feasibility Study

**DOI:** 10.3390/bios13070717

**Published:** 2023-07-07

**Authors:** Magnus Falk, Carolin Psotta, Stefan Cirovic, Lars Ohlsson, Sergey Shleev

**Affiliations:** Biomedical Science, Faculty of Health and Society, and Biofilms Research Center, Malmö University, 205 06 Malmö, Sweden; magnus.falk@mau.se (M.F.); carolin.psotta@mau.se (C.P.); stefan.cirovic@mau.se (S.C.); lars.ohlsson@mau.se (L.O.)

**Keywords:** electronic tongue, differential pulse voltammetry, principial component analysis, authentic human saliva, SARS-CoV-2

## Abstract

An electronic tongue is a powerful analytical instrument based on an array of non-selective chemical sensors with a partial specificity for data gathering and advanced pattern recognition methods for data analysis. Connecting electronic tongues with electrochemical techniques for data collection has led to various applications, mostly within sensing for food quality and environmental monitoring, but also in biomedical research for the analyses of different bioanalytes in human physiological fluids. In this paper, an electronic tongue consisting of six electrodes (viz., gold, platinum, palladium, titanium, iridium, and glassy carbon) was designed and tested in authentic (undiluted, unpretreated) human saliva samples from eight volunteers, collected before and during the COVID-19 pandemic. Investigations of 11 samples using differential pulse voltammetry and a principal component analysis allowed us to distinguish between SARS-CoV-2-free and infected authentic human saliva. This work, as a proof-of-principle demonstration, provides a new perspective for the use of electronic tongues in the field of enzyme-free electrochemical biosensing, highlighting their potential for future applications in non-invasive biomedical analyses.

## 1. Introduction

During the coronavirus disease 2019 (COVID-19) pandemic [1], numerous (bio-)sensors have been developed for the detection of severe acute respiratory syndrome coronavirus 2 (SARS-CoV-2) based on different readout techniques and sample matrices utilised [2,3,4,5,6,7,8,9]. On the one hand, current reports in the literature indicate that electrochemical (bio-)sensors were tested to a minimal extent in authentic (undiluted and unpretreated) human physiological fluids, and tests were conducted using multi-step detection protocols. On the other hand, these (bio-)devices should be capable of operating in authentic complex matrices, including human physiological fluids like saliva [10,11,12], due to their high selectivity and specificity [13,14,15]. 

An electronic tongue (e-tongue) based on an electrochemical readout technique with a multivariate data analysis for pattern recognition can be used to achieve the required selectivity and specificity for direct operation in authentic human physiological fluids. An e-tongue is a powerful tool in sensor research, which utilises an array of non-selective, highly stable, and cross-sensitive sensors together with a suitable pattern recognition algorithm [16,17,18]. A principal component analysis (PCA) is one of the pattern recognition methods used for e-tongue signal processing [18,19,20], and it constitutes a method to simplify, disentangle, and reduce (big) data sets without losing essential information about the samples and the variables of which [20,21,22,23,24].

So far, e-tongues have been employed for various applications, e.g., the analysis of foodstuff [25,26], beverages including wine [27,28,29,30], and chemicals. E-tongues have also found a use in environmental monitoring [31,32], in the assessment of pharmaceutical formulations [33,34,35], and for the analysis of physiological fluids, like saliva [36,37]), sweat [38], and dialysate fluid [39]. However, regarding COVID-19 detection, only one report was found in the literature. The detection was reliant on a colourimetric readout e-tongue and PCA to monitor the profile concentrations of chemical markers in a specially pre-treated saliva supernatant [40]. 

Thus, to the best of our knowledge, no e-tongue-based (bio-)sensor for SARS-CoV-2 detection operating in unadulterated human physiological fluids has been reported so far, even though some reports regarding voltametric (including differential pulse voltammetry, DPV [41,42]) SARS-CoV2 detection in phosphate-buffered saline and nasopharyngeal samples do exist in the literature. In this paper, we describe a voltametric, DPV-based e-tongue for the distinction of SARS-CoV-2-free or infected authentic human saliva, employing PCA for pattern recognition. The developed e-tongue was able to tell apart virus-free and infected saliva samples without special sample pre-treatment or dilution, as demonstrated below.

## 2. Materials and Methods

### 2.1. Chemicals

All chemicals used were of an analytical grade, obtained from Sigma Aldrich (St. Louis, MO, USA). For electrochemical measurements (vide infra) in buffer, a 10 mM phosphate buffer, pH 6.9, containing 10 mM of NaCl (PBS) was used as an electrolyte.

### 2.2. Volunteers

Before the initiation of this study, the Swedish Ethical Review Authority (Etikprövningsmyndigheten) approved this research (DNR 2019-01741 and 2022-03536-01) based on two written ethical applications prepared following the World Medical Association Declaration of Helsinki “Ethical Principles for Medical Research Involving Human Subjects”. All activities were performed following guidelines and regulations provided by the Swedish Ethical Review Authority. All participants were initially informed that they will take part in a research process, the results of which might be published. They were also informed that the objective of the research was to explore new technologies for SARS-CoV-2 diagnostics. Participation was voluntary and participants were informed that they could leave this study anytime. Written information on the research process was also provided and participants were asked to sign an informed consent sheet. All materials collected were marked by a code, kept confidential, and could be accessed only by the research team members.

### 2.3. Saliva Sample Collection

Eleven saliva samples from 8 apparently healthy volunteers were collected into 10 mL VWR sample tubes from Avantor (Gliwice, Poland) via passive drool before and after lunch, stored at −25 °C, and thawed at room temperature directly before use.

### 2.4. Analysis of Saliva Samples with Real-Time Quantitative Polymerase Chain Reaction

A master mix COVID-19 real-time quantitative polymerase chain reaction (RT-qPCR) kit LiliFTM (Supplementary Table S1) was purchased from Biotecnologia iNtRON (Gyeonggi-do, Republic of Korea). Invitrogen RNase Zap Wipes and PCR plates, 96-well, Finn tip Filters, 10 µL, 200 µL, and 1000 µL, sterile, were from ThermoFisher Scientific (Waltham, MA, USA). For RT-qPCR, sample preparation handling Fisher pipettes from ThermoFisher Scientific and a microcentrifuge from Dlab (Los Angeles, CA, USA) were used. To perform RT-qPCR, the reagents listed in Supplementary Table S1 were used according to the manufacturer’s protocol. For the analysis of clinical samples, a three-step cycling method was used (Supplementary Table S2). A Light Cylinder 480 II Software version 1.5 PCR system from Roche (Basel, Switzerland) was exploited. Before conducting RT-qPCR, RNA was extracted using a Patheo Gene-spinTM DNA/RNA Extraction Kit from Biotecnologia iNtRON according to the manufacturer’s protocol. All reagents, their corresponding volumes, basic parameters, etc., used during the RT-qPCR analysis are listed in Supplementary Tables S3 and S4.

### 2.5. Electrochemical Measurements

Electrochemical measurements were performed using a μAutolab Type III/FRA2 potentiostat/galvanostat from Metrohm Autolab B.V. (Utrecht, The Netherlands). A 2 mL electrochemical cell with a standard three-electrode configuration with an Ag|AgCl|3 M KCl reference electrode and a platinum mesh counter electrode was used. Prior to measurements, the electrodes were gently polished with 0.05 μm of aluminium oxide powder from Struers (Westlake, OH, USA). After that, they were cleaned electrochemically with cycling in 0.5 M of H_2_SO_4_, following the procedure described in Ref. [43]. All electrochemical measurements were performed twice using DPV. A potential range from −0.6 to 0.8 V and a step potential of 1.98 mV with a 25 mV amplitude and 0.1 s interval time were applied, recording 706 individual data points for each DPV measurement using 2 mL of PBS or a saliva sample.

### 2.6. E-Tongue Fabrication

An e-tongue was designed using commercially available electrodes from BASi^®^ (West Lafayette, IN, USA) made of gold (Au) and platinum (Pt) (2 mm in diameter), as well as palladium (Pd), titanium (Ti), iridium (Ir), and glassy carbon (Gc) (3 mm in diameter).

### 2.7. Data Treatment using PCA

PCA was performed using SPSS Statistics from IBM Corp. (Armonk, New York, NY, USA) as an unsupervised tool for dimension reduction to qualitatively analyse the voltammetric responses. Prior to the analysis, the data sets for responses from the six working electrodes were merged into one data set of 4236 data points and analysed together with each repeat measurement and each fluid, with the responses standardized to remove effects of different electrode sizes by having each variable (response from each electrode) scaled to unit variance and mean centred. After pre-processing, PCA of samples was performed, extracting all factors with an eigenvalue greater than 1, without any additional factor rotation.

## 3. Results and Discussion

### 3.1. Saliva Samples and Their RT-qPCR Analysis

First, authentic saliva samples—sample volumes ca. 5 mL (11 in total, Table 1) from eight volunteers, four women and four men—were collected via passive drool into 10 mL VWR tubes before the COVID-19 pandemic (in October 2019) and during the COVID-19 pandemic, viz., in January–February 2022, when the SARS-CoV-2 infection rate was the highest in Sweden, as well as in June 2022, when the rate was negligible (Supplementary Figure S1). More specifically, two “pre-pandemic” samples were collected from the first volunteer before and after lunch (samples 1 and 2), whereas all “pandemic” samples (nine in total) were collected from eight volunteers before lunch (samples 3–11, Table 1), including two samples from the first volunteer.

The standard testing methods for SARS-CoV-2 detection, e.g., the identifying virus antibodies, like IgG or IgM, the viral protein/antigen, or the viral RNA, are based on various sample collection techniques, and most of them are either uncomfortable or invasive, i.e., the nasal swab, throat swab, nasopharyngeal swabs, bronchoalveolar lavage, or blood donation sampling [44,45,46]. For the identification of SARS-CoV-2, the current laboratory techniques are RT-PCR and RT-LAMP but also include microfluidic devices for multiplexing, microarrays, and lateral flow assays based on a colourimetric readout [44,45]. RT-PCR is a time-consuming and expensive process, which requires a well-equipped laboratory and skilled personnel [44,45,47].

**Figure 1:**
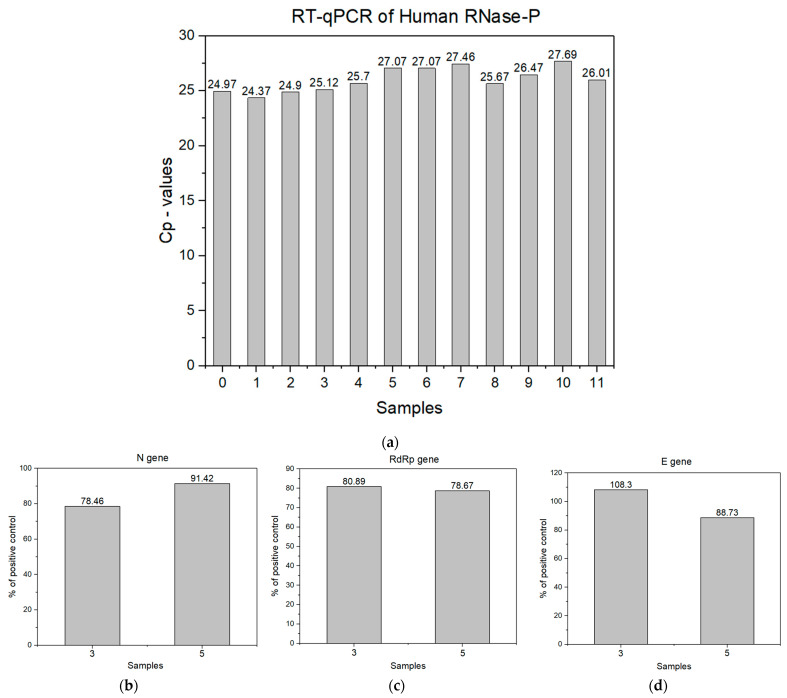
RT-qPCR analysis. (**a**) Cp values from RNase-P sample extraction (sample 0—positive control). (**b**–**d**) The bar graphs depict the calculated concentrations of N, RdRp, and E genes in a percent of the positive control, using the obtained Cp values.

Despite the PCR disadvantages (vide supra), the second step in this work was a RT-qPRC analysis, the COVID-19 diagnostic gold standard [48], starting from RNA extraction from all saliva samples. The efficacy of RNA extraction from human saliva was assessed using ribonuclease-P (RNase-P) as an internal control for validation of the PCR test. RNase-P is an essential enzyme that catalyses the removal of the 5′ leader from precursor transfer RNAs. The ribonucleoprotein (RNP) form of RNase-P is present in all live form domains of [49,50] and comprises a single catalytic RNA (ribozyme) and a variable collection of protein co-factors. Thus, human RNase-P is highly recommended by the Centers for Disease Control and Prevention (USA) as an internal control for human clinical specimens and should be used to confirm the validity of all test reactions during SARS-CoV-2 detection [51]. In RT-qPCR, Cp values above 35 are considered unacceptable since, at high Cp values, the signal is weak, and the data become less reliable, with an increased likelihood of false-positive or false-negative results. In addition, a high Cp value suggests that the sample may contain low levels of target RNA, which may necessitate additional validation of the results. The obtained data from RT-qPCR for human RNase-P are in good agreement with the manufacturer’s protocol, RT-PCR Kit LiliFTM by Biotecnologia iNtRON, viz., the obtained crossing point (Cp values) for all 11 samples that are within the acceptable range, as illustrated in Figure 1a.

To detect the presence of SARS-CoV-2 in the samples, analyses of three genes, viz., N, RdRp, and E genes, were performed. These gene analyses as markers for detecting new coronaviruses are strongly recommended by the World Health Organisation [52], confirming that SARS-CoV-2 belongs to the beta coronavirus family, like SARS-CoV and MERS-CoV [53]. To analyse the presence of amplified SARS-CoV-2 specific genes in the samples, the obtained Cp values were compared with that of the positive control, as suggested by the manufacturer. The RT-qPCR analysis revealed that among 11 samples collected from the volunteers and used in this work, only 2 samples were SARS-CoV-2-positive, viz., samples 3 and 5, as evident from Figure 1b–d. It is important to emphasise that the three morning samples from the first volunteer correspond to the pre- (sample 1) and intrapandemic (samples 3 and 4) phases, with samples 3 and 4 reflecting SARS-CoV-2 infected and infection-free saliva, respectively. This is quite a unique situation in a biomedical analysis, which allows us to investigate authentic saliva from one individual before (sample 1) and during COVID-19 (sample 3), as well as after recovery from the disease (sample 4).

### 3.2. Characterisation of the E-Tongue in PBS and Healthy (Pre-COVID-19) Saliva Samples

**Figure 2:**
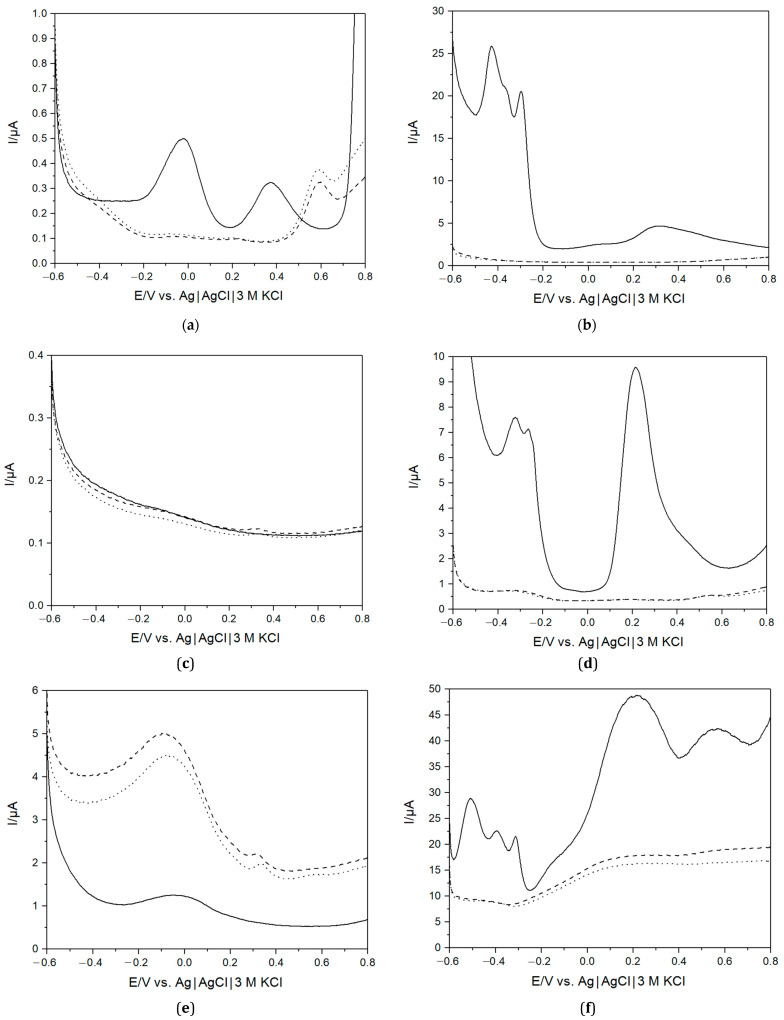
Differential pulse voltammograms of PBS (solid line) and saliva collected before lunch (dotted line, sample 1) and after lunch (dashed line, sample 2) using Au (**a**), Pt (**b**), Ti (**c**), Pd (**d**), Gc (**e**), Ir (**f**) electrodes.

To investigate the viability of using an e-tongue to distinguish between different samples, saliva collected before and after lunch from one apparently healthy volunteer during October 2019, i.e., before the COVID-19 pandemic (the SARS-CoV-2 free nature of these samples was additionally confirmed with RT-qPCR, vide supra), was used. The e-tongue composed of Au, Pt, Pd, Ir, Ti, and Gc electrodes was chosen due to the stability and measurement reproducibility of the electrode materials [54]. Typical current responses for the electrodes in PBS and saliva recorded using DPV are displayed in Figure 2. The electrochemical behaviour of these electrodes in PBS at neutral pH is well studied and understood. On polycrystalline Au, large currents at minimal and maximal redox potentials, i.e., below -0.6 V and above 0.8 V, could be attributed to H_2_ evolution [55] and H_2_O electro-oxidation [56], respectively. The peak at about −0.05 V corresponded, in all likelihood, to O_2_ reduction to H_2_O_2_ [57], whereas the peak at ca. 0.38 V reflected complex Au/phosphate/chloride electrochemistry (Figure 2a). On Pt, Pd, and Ir electrodes, well-pronounced H_2_ evolution [55] and O_2_ reduction reactions [57] were observed below and above −0.2 V and 0 V, respectively (Figure 2b,d,f). Taking into account the electrochemical pre-treatment of all the electrodes in H_2_SO_4_ (vide supra), the visible peak at −0.05 V on the voltammogram of the Gc electrode could be attributed to electroactivation of the material with the formation of oxygenous functionalities on the surface, e.g., quinone-type groups (Figure 2e), whereas the Ti electrode was almost electrochemically inactive in the used potential range, viz., −0.6 V–+0.8 V vs. Ag|AgCl|KCl (3M), due to the presence of a defective TiO_2_ surface film (Figure 2c).

A noteworthy difference was observed when comparing measurements in saliva and the buffer. In the former, none of the peaks attributable to H_2_ evolution and O_2_ reduction were observed in the case of the Pt group metal electrodes (Figure 2b,d,f). A provisional explanation could be that the many compounds present in saliva [58] may adsorb on the surface and change the electrocatalytic properties of these materials [59]. Similar changes were also observed in voltammograms of Au electrodes, but also a new, well-pronounced peak appeared at ca. 0.6 V (Figure 2a). The origin of this peak is unclear; however, based on the presence of slowly growing current shoulders at a potential above 0.4 V on the voltammograms of the Pt and Pb electrodes (Figure 2b,d), similarly noticed on Pt in blood [57], one could suggest that this peak corresponded to the oxidation of some electrochemically active compounds, like ascorbate, present in saliva [58]. Last but not least, it is important to mention a small peak at a potential of about 0.3 V (Figure 2e) in the voltammograms of the Gc electrode submerged in saliva (vide infra).

A difference is also observed between the two different saliva samples. Since saliva contains many different compounds that would affect the response observed with DPV, a large difference between saliva and the buffer is expected. However, it is also clear that compositional changes between different saliva samples can be observed with the e-tongue. The saliva produced by the salivary glands consists of about 98% water [10,58,60,61]. However, despite its watery nature, saliva is a complex biological fluid that contains a diverse range of compounds and has multiple functions in the oral cavity, e.g., playing an important role in the immune system and digestion [10,58,60,61]. Saliva contains a wealth of organic substances, such as enzymes, hormones, cytokines, immunoglobulins, mucins, etc., and metabolites and antioxidants (e.g., urea, amino acids, uric acid, lactic acid, ascorbic acids), as well as inorganic compounds, such as potassium and sodium ions, the chloride ion, bicarbonate, etc. [62,63,64]. Indeed, by switching from PBS to the complex physiological fluid, the obtained voltammograms were unprecedentedly changed. The composition and proportion of substances in saliva dynamically covaries with the physiological state and can change, e.g., due to diet or disease, and can explain the observed differences between the measured saliva samples.

**Figure 3:**
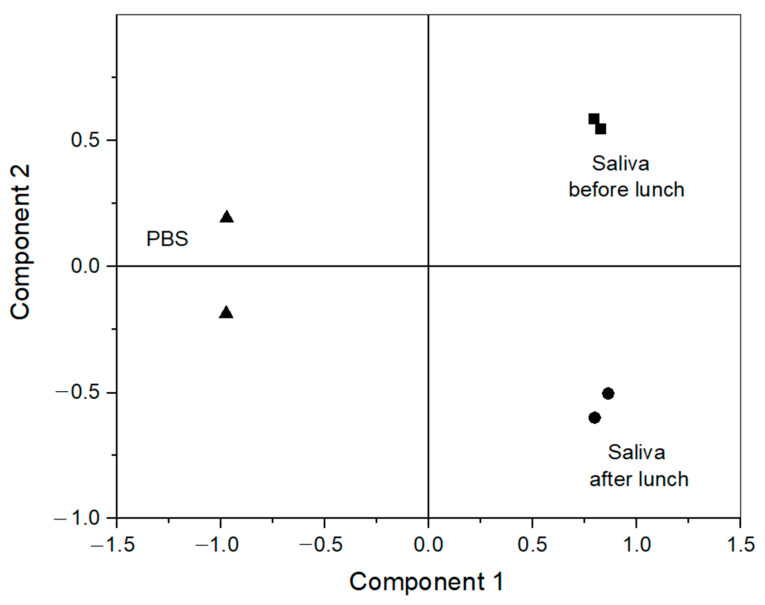
PCA score plot of two different repeat measurements using the e-tongue in PBS (triangles) and saliva collected before (squares, sample 1) and after (circles, sample 2) lunch. The explained variance of PC 1 (76.3%) and PC 2 (22.2%).

PCA was used to characterise the response recorded from the different electrodes in PBS and saliva samples. To perform the analysis, the data from each electrode were merged by combining the different data sets in a large data set of the size 6 × 4236 (three different fluids each tested twice, using six different working electrodes, each recording 706 data points). Discrimination of the samples was possible by studying the score plots. In total, two different principal components (PCs) were extracted, all with absolute eigenvalues larger than 1, explaining a total of 98.5% of the variance in the samples (76.3% and 22.2% for PC 1 and 2, respectively). The PCs are illustrated in Figure 3, and the individual loading score can be found in Supplementary Table S5. As expected, based on the first component, the saliva samples are clearly separate from PBS, but the difference between the different saliva samples is also clear from the second component. This shows that the e-tongue can also be used to distinguish between different saliva samples. However, it should be noted that without an additional chemical analysis performed on the samples, it is impossible to determine the contents based only on the response of the e-tongue, since the samples consist of several redox-active compounds and various ions, as well as other compounds that could affect the response, e.g., via adsorption. The compositional difference between the samples was not analysed with additional methods.

### 3.3. Investigation of SARS-CoV-2-Free and Infected Saliva Samples using PCR and e-Tongue

**Figure 4:**
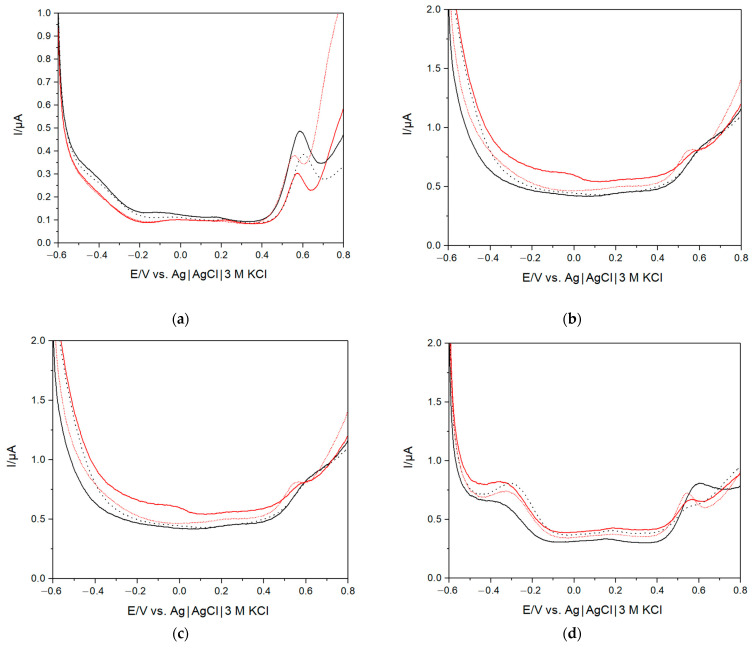

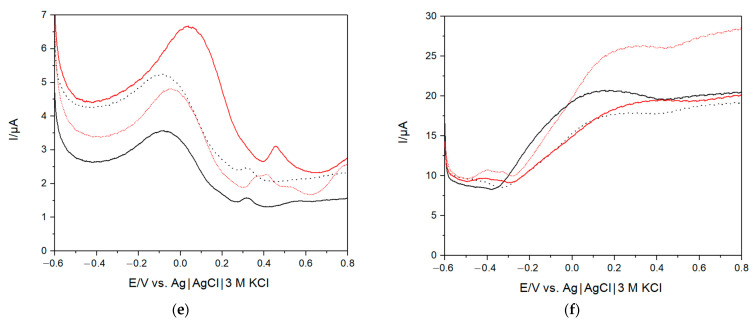
Differential pulse voltammograms of two different SARS-CoV-2-negative (solid black—sample 1, dotted black—sample 9) and two different SARS-CoV-2-positive (solid red—sample 3, short dotted red—sample 5) saliva samples using Au (**a**), Pt (**b**), Ti (**c**), Pd (**d**), Gc (**e**), Ir (**f**) electrodes.

Nine SARS-CoV-2-free and infected saliva samples were also analysed using the assembled e-tongue, exploiting the same principle as described in Section 3.2. Differential pulse voltammograms for two SARS-CoV-2-negative and two positive saliva samples are shown in Figure 4 (differential pulse voltammograms for all the samples are presented in Supplementary Figure S2). In general, all different samples produced slightly different responses, which is expected due to the varying composition of saliva from different volunteers. A few distinguishing features can be observed when looking for possible differences in the response between SARS-CoV-2-negative and -positive samples. For instance, for the Au electrode, a slight shift of the redox peaks assigned to electro-oxidation of antioxidants could be observed (Figure 4a), whereas for the GC electrode, a previously noted small peak shifted from around +0.3 V to +0.4 and +0.45 V (Figure 4e). Moreover, in the case of one infected sample, a quite pronounced electro-oxidative process at potentials above +0.6 V could be seen. Nevertheless, due to the large variability of the samples, it is challenging to separate SARS-CoV-2-negative from -positive samples based on the recorded differential pulse voltammograms only.

**Figure 5:**
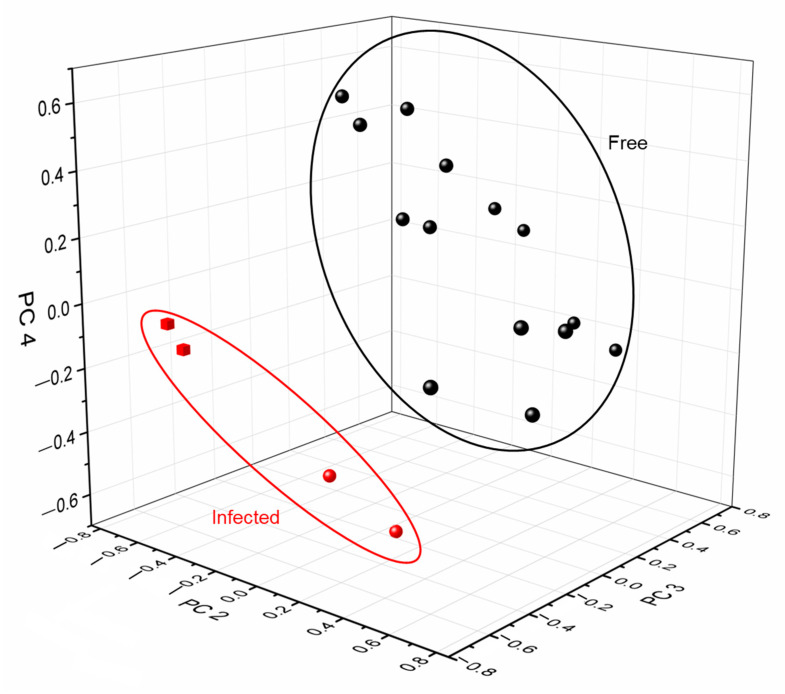
PCA score plot of two different repeat measurements for each sample using the e-tongue in seven SARS-CoV-2-negative (samples 4, 6–11, black spheres) and two SARS-CoV-2-positive (sample 3, red cubes, and sample 5, red spheres) saliva samples. The explained variance of PC 2 (21.3%), PC 3 (15.9%), and PC 4 (12.9%).

PCA was performed to enable the differentiation of SARS-CoV-2-negative and -positive samples by combining the different data sets in a large data set of the size 18 × 4236 (nine different saliva samples each tested twice, using six different working electrodes, each recording 706 data points). The results of the analysis of the combined response from the electronic tongue are shown in Figure 5 and Supplementary Table S6, where the individual loadings for each sample on the different PCs are shown. In total, four PCs were extracted, explaining 84.5% of the total variance in the measured samples, whereas the individual components explained 34.4%, 21.3%, 15.9%, and 12.9%, respectively (PC 1, PC 2, PC 3, and PC 4). A large observed variation between the saliva samples was also seen in the different sample loadings, particularly reflected in the first PC, which explains the most of the sample variation, where repeat measurements were very similar, but different samples were distinguishable from each other with large differences observed in the loadings between samples. The differences can be related to sample variability, relating to factors such as possible contamination by, e.g., food or medications, variability in the salivary flow rate affecting analyte concentrations, oral health affected by, e.g., inflammation, as well as unique individual compositional differences caused by, e.g., genetics or general health conditions. However, as shown in Figure 5, looking at the last three PCs, SARS-CoV-2-negative samples could be separated from positive samples, where the positive samples showed a zero or negative loading on all three components. This indicates that there may be a compositional change in positive samples, distinguishable by the electronic tongue. Salivary metabolites have previously been used to detect diseases and explore a health status, where changes in the metabolic profile due to the activity of the immune system in the oral cavity could be identified [10,11,58,59,65]. Recent studies have focused on compositional changes of saliva related to SARS-CoV-2, where the viral infection has been shown to induce distinctive metabolic derangement [14,40,66,67,68]. The general characteristics of healthy saliva samples were altered due to changing amino acid metabolism (downregulation of major amino acids like histidine [14]), increased immune system activity [14,66], and transformed molecular changes [14,66,67,68,69]. Among specific changes, the levels of saliva metabolites (sphingosine and kynurenine) were significantly different between SARS-CoV-2-infected and non-infected samples [68]. Increased gamma glutamyl-transferase activity and decreased total esterase activity were also observed in the saliva of SARS-CoV-2 patients [66]. The separation of SARS-CoV-2-positive and -negative samples enabled by the electronic tongue herein investigated could thus be explained by compositional changes in saliva upon infection. However, it should be noted that the current sample size was very small, and further studies would be needed to verify the results.

**Figure 6:**
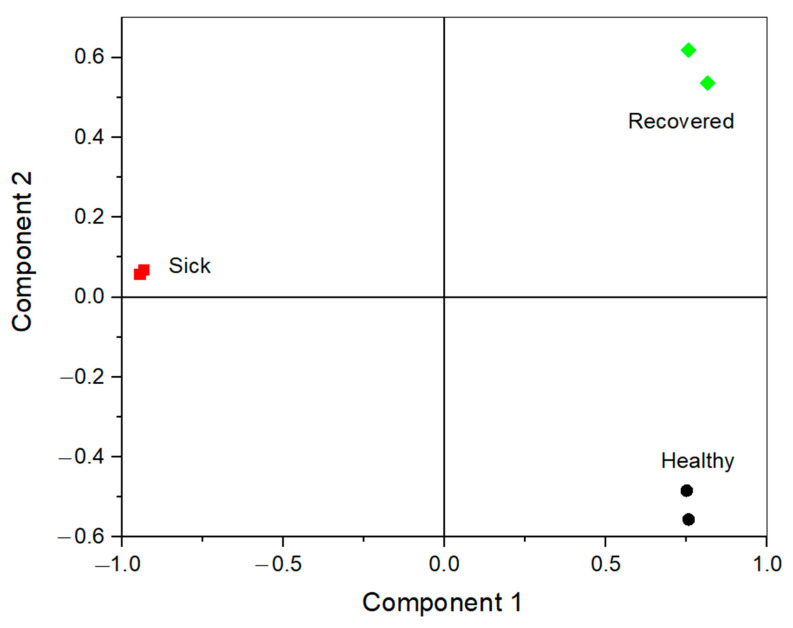
PCA score plot of two different repeat measurements using the e-tongue in two SARS-CoV-2-negative (sample 1, healthy, black dot, and sample 4, recovered, green rhombus) and one SARS-CoV-2-positive (sample 3, infected, red square) saliva samples, collected in the morning from the same volunteer. The explained variance of PC 1 (69.0%) and PC 2 (20.3%).

Three different morning saliva samples collected from the same volunteer, i.e., healthy, infected, and recovered (samples 1, 3, and 4, respectively), were analysed with DPV using the e-tongue. Certain differences between the three voltammograms were observed for all six electrodes (Supplementary Figure S3). For instance, higher currents overall were registered on Pt, Ti, and Gc electrodes. Additionally, at the Gc electrode, the peak at ca. 0.45 V was more prominent and also shifted in the oxidative direction (Supplementary Figure S3e); at the Au electrode, the current increased sharply at very positive potentials (Supplementary Figure S3a), which might reflect much higher concentrations of antioxidants in infected saliva compared to the fluid from the healthy or recovered individual. Nevertheless, due to the large variability of the samples, it is hard or even impossible to identify SARS-CoV-2-positive samples based on the recorded differential pulse voltammograms only. Thus, PCA was performed by combining the different data sets in a large data set of the size 6 × 4236 (three different saliva samples each tested twice, using six different working electrodes, each recording 706 data points). The main results of the PCA are shown in Figure 6, where two PCs were extracted, explaining 89.3% of the total variance in the measured samples, whereas the individual components explained 69.0% and 20.3%, respectively (PC 1 and PC 2). While the separate morning saliva samples collected on different days show a large variation, which is reflected in the different loadings of the samples on PC 2, the SARS-CoV-2-positive sample was distinctly different from the healthy and recovered samples in the PCA. This difference is reflected in the first PC, which also explained the majority of the variance observed between the samples, where the negative (healthy and recovered) samples have a similar loading opposite the positive samples.

## 4. Conclusions

In this paper, an e-tongue fabricated from six different electrodes was tested in PBS and authentic human saliva collected from eight different volunteers before and during the COVID-19 pandemic. A clear difference in the electrochemical response was observed when the device operated in the simple buffer compared to the physiological fluid, reflecting the complex composition of human saliva. Certain differences between differential pulse voltammograms of the multi-sensor operating in SARS-CoV-2-positive and -negative saliva samples, which were confirmed with RT-qPCR, were also identified. Also, some differences in the electrochemical behaviour of the e-tongue were registered when the device operated in saliva samples from one volunteer collected at different time points, i.e., healthy, COVID-19 infected, and recovered. The exploitation of PCA allowed us to distinguish saliva samples from COVID-19-positive and -negative volunteers using unpretreated and undiluted human physiological fluid. Taking into account the previous exploitation of e-tongues for the analysis of human sweat and urine, a new perspective for the use of these devices in the field of enzyme-free electrochemical biosensing is emerging, highlighting their potential for future applications in non-invasive medical diagnostics.

## Figures and Tables

**Table 1:** Basic information regarding volunteers and samples used in this study.

Volunteer №	Sample №	Date of Collection	PCR Test	Comments
1	1	10/2019	−	Before lunch
1	2	10/2019	−	After lunch
1	3	01/2022	+	Before lunch
1	4	06/2022	−	Before lunch
2	5	02/2022	+	Before lunch
3	6	01/2022	−	Before lunch
4	7	01/2022	−	Before lunch
5	8	02/2022	−	Before lunch
6	9	02/2022	−	Before lunch
7	10	02/2022	−	Before lunch
8	11	02/2022	−	Before lunch

## Data Availability

The data that support the findings of this study are available from the corresponding author upon reasonable request.

## Supplementary Materials

The following supporting information can be downloaded at: https://www.mdpi.com/article/10.3390/bios13070717/s1, Figure S1: Monthly report of SARS-CoV-2-infected patients in Sweden; Figure S2: Differential pulse voltammograms of the e-tongue recorded in non-infected saliva samples; Figure S3: Differential pulse voltammograms of the e-tongue recorded in SARS-CoV-2-free and SARS-CoV-2-infected saliva samples; Table S1: COVID-19 real-time RT-PCR kit; Table S2: Three-step cycling method for RT-qPCR; Table S3: LightCycler^®^ 480 real-time PCR system detection formats; Table S4: The list of RT-qPCR components with their respective volumes; Table S5: PC score values for saliva and buffer samples; Table S6: PC score values for SARS-CoV-2-negative and SARS-CoV-2-positive saliva samples.
