# Supplementary material for: Electronic Tongue for Direct Assessment of SARS-CoV-2-Free and Infected Human Saliva—A Feasibility Study
Source: Biosensors (Basel). 2023 Jul 7;13(7):717. doi: 10.3390/bios13070717 (PMC10377364; doi:10.3390/bios13070717)
Supplement: Supplementary file 1 [file biosensors-13-00717-s001.zip › biosensors-2422029-supplementary.pdf]

Supplementary Materials

Electronic Tongue for Direct Assessment of SARS-CoV-2-Free and Infected Human Saliva—A Feasibility Study

Magnus Falk, Carolin Psotta, Stefan Cirovic, Lars Ohlsson and Sergey Shleev *

Biomedical Science, Faculty of Health and Society, and Biofilms Research Center, Malmö University, 205 06 Malmö, Sweden
* Correspondence: sergey.shleev@mau.se (SS)

Sample Collection

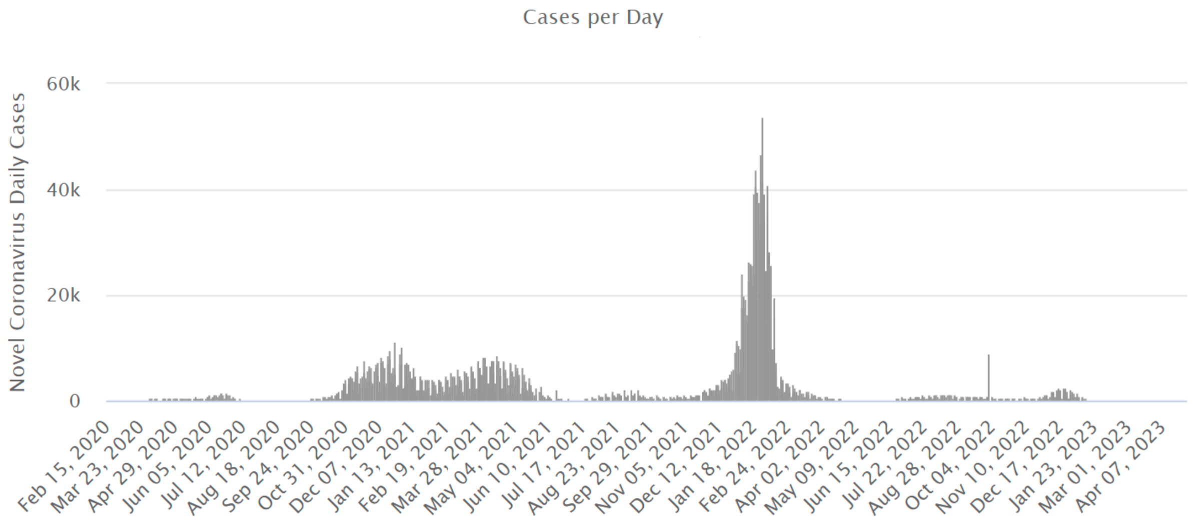

Supplementary Figure S1. Monthly report of SARS-CoV-2-infected patients in Sweden.

Analysis of saliva samples with real-time quantitative polymerase chain reaction (RT-qPCR)

Multiplex PCR enables the simultaneous detection of multiple targets in a single reaction well, with a different pair of primers and probes for each target. In this study, PCR products were monitored using an hydrolysis probe assay. Hydrolysis probe assays are in fact 5′ nuclease assays, because the 3′end of the hydrolysis probe is phosphorylated and cannot be extended during a PCR reaction. Every single probe contains two labels, a fluorescence reporter dye, and a fluorescence quencher dye, which are very close to each other. When the probe is intact, the quencher dye is close enough to the reporter dye to suppress or block the reporter fluorescent signal via a fluorescence resonance energy transfer (FRET). During PCR, the 5′-nuclease activity of the polymerase cleaves the hydrolysis probe and separates the reporter from the corresponding quencher. When the probe is cleaved, the reporter can no longer be quenched and emits a fluorescence signal. The fluorescence is measured with one or two channels of the Light Cycler®. Each channel measures different dyes depending on their colour, excitation, and detection of light in nm. The following reporter dyes are used in this research: Fluorescein amidite (FAM) and Hexachloro-fluorescein (HEX) (Supplementary Table S3).

Supplementary Table S1. COVID-19 real-time RT-PCR kit.

No.	Components	Ingredients
1	2X RT-PCR	dNTP (dATP, dTTP, dGTP, dCTP) Hot Start Taq DNA Polymerase, Reverse Transcriptase
2	RdRp/E Detection solution	RdRp Primer/probe<br>E Primer/probe
3	N/RNaseP Detection solution	N Primer/Probe<br>RNase-P Primer/probe
4	Positive Control	Non-infectious plasmid DNA (microbial) containing betacoronavirus E gene / COVID-19 RdRp gene / COVID-19 N gene / human RNase-P gene sequences
5	DNase/RNase Free Water	No template control, 100% DNase/RNase Free Water

Supplementary Table S2. Three-step cycling method for RT-qPCR.

Cycles	Temperature (℃)	Time (s)	Notes
1	95	120 s	Polymerase activation
40	95	5 s	Denaturation
1	60-65	10 s	Annealing
1	72	5-20 s	Extension

Supplementary Table S3. LightCycler® 480 real-time PCR system detection formats.

Applications	Assay detection format	Dyes/genes	Excitation (nm)	Detection (nm)
**Quantification**	Dual Colour Hydrolysis Probes/UPL Probes	FAM/RdRp and N gene	465	510
**Quantification**	Dual Colour Hydrolysis Probes/UPL Probes	HEX/E gene and RNase-P	533	580

To extract viral RNA, the following procedure was used. In total, 150 µl of diluted saliva with ddH_2O 1:1 was transferred into a 1.5 ml microcentrifuge tube, 300 µl of a Lysis Buffer was added, mixed with vortexing for 15 sec, and incubated at room temperature (15-25 °C) for 10 min, 300 µl of a binding buffer was added, and the final solution was completely mixed with gentle vortexing. The mixed solution was placed on a spin column in a provided 2 ml collection tube. The lysates were loaded on the column and centrifuged at 13,000 rpm for 1 min. The solution in the collection tube was discarded and the column was placed in the same 2 ml collection tube, and 500 µl of washing buffer A was added to the column and it was centrifuged for 1 min at 13,000 rpm. The solution in the collection tube was discarded again and the spin column was returned to the same 2 ml collection tube. In total, 500 µl of washing buffer B was applied to the column and the column was centrifuged for 1 min at 13,000 rpm. Washing buffer B was supplied as a concentrate. Before using it for the first time, 40 mL of ethanol (96~100%) was added to 10 ml of the buffer as indicated on the bottle. The solution in the collection tube was discarded, and the spin column back in the same 2 ml collection tube was placed and centrifuged for 1 min at 13,000 rpm. It is important to dry the membrane since residual ethanol may interfere with downstream reactions. The column was placed in an RNase-free 1.5 ml microcentrifuge tube, and 60 µl of the elution buffer was added directly onto the spin column membrane, incubated at room temperature for 1 min,

and then centrifuged for 1 min at 13,000 rpm. In the final step, the eluted solution was placed in a -80 °C freezer. In total, 5 µl of the eluted solution was used as a template for RT-qPCR.

Supplementary Table S4. The list of RT-qPCR components with their respective volumes.

**Component**	RT-PCR master mix	RdRP/E	N/RNase-P	Sample	Positive control	Negative control DNase/RNase-free water
**Volume**	10 µl	5 µl	5 µl	5 µl	10 µl	10 µl

Electrochemical measurements and PCA

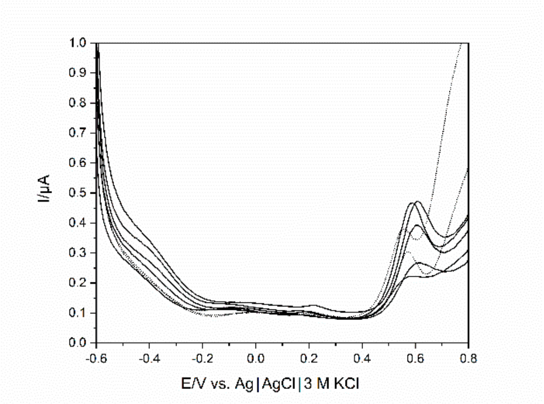

(**a**)

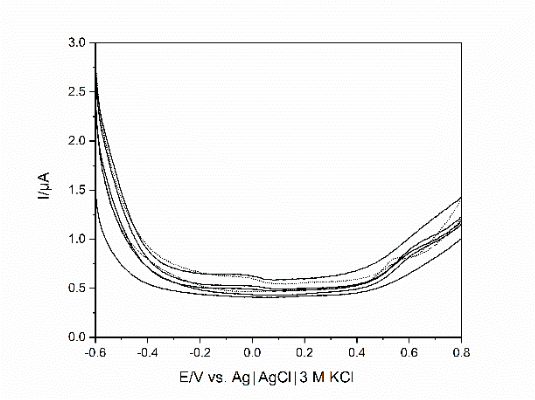

(**b**)

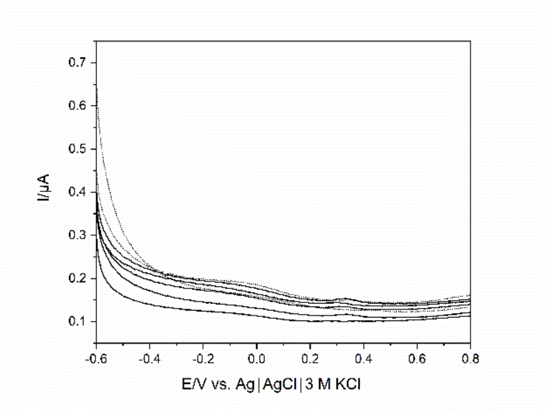

(**c**)

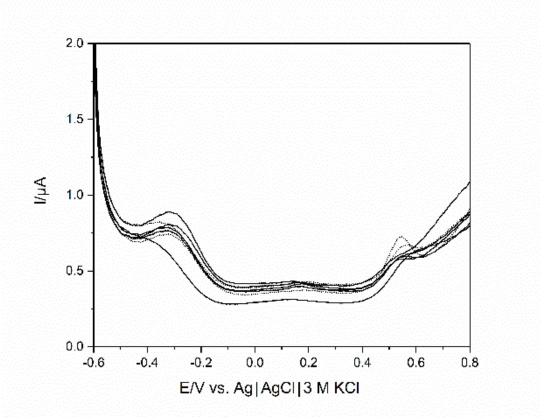

(**d**)

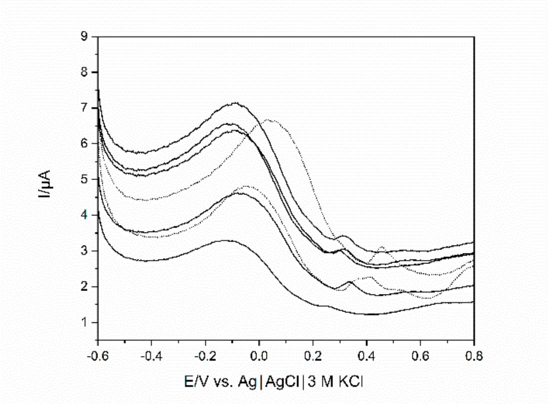

(**e**)

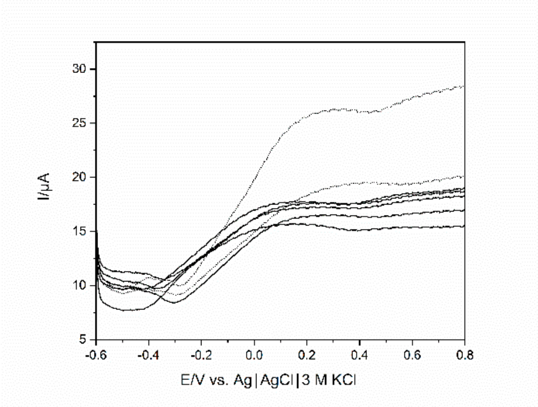

(**f**)

Supplementary Figure S2. Differential pulse voltammograms of the e-tongue recorded in non-infected saliva samples 4, 6-11: Au (**a**), Pt (**b**), Ti (**c**), Pd (d), Gc (**e**), Ir (**f**).

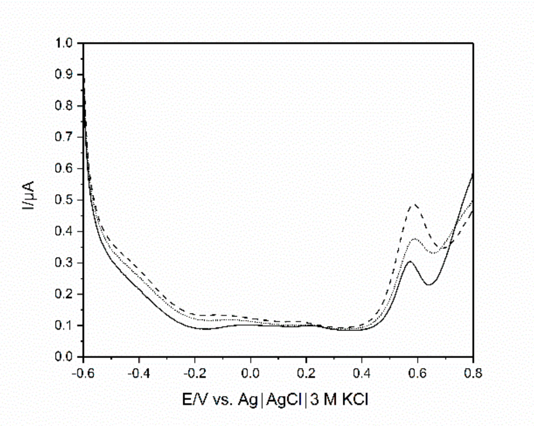

(**a**)

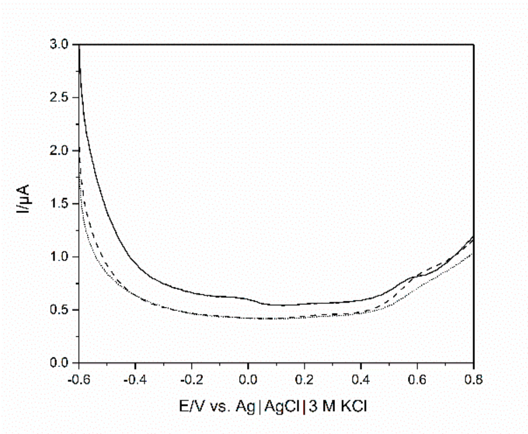

(**b**)

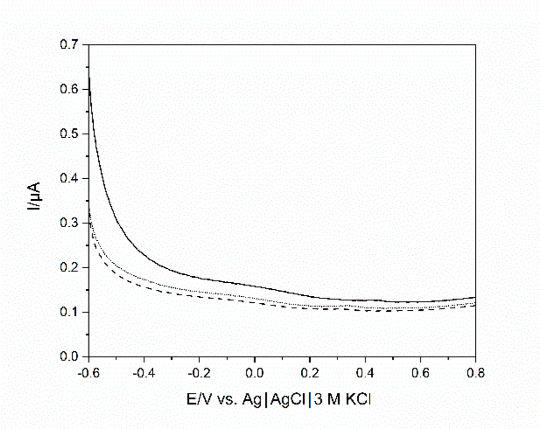

(**c**)

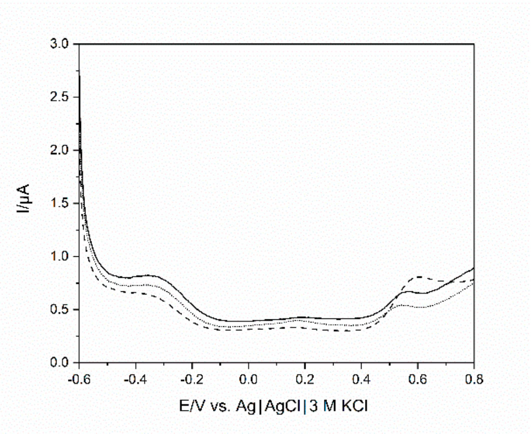

(**d**)

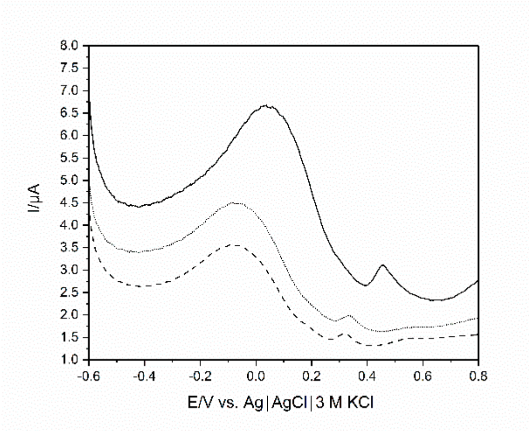

(**e**)

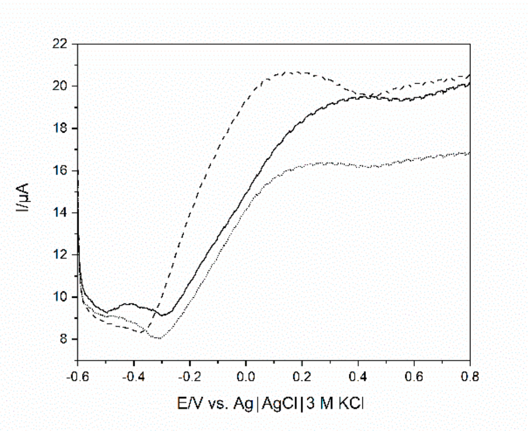

(**f**)

Supplementary Figure S3. Differential pulse voltammograms of the e-tongue recorded in SARS-CoV-2-free (sample 1, healthy volunteer, dotted curve, and sample 4, cured volunteer, dashed curve) and SARS-CoV-2-infected (sample 3, sick volunteer, solid curve) saliva samples: Au (**a**), Pt (**b**), Ti (**c**), Pd (d), Gc (**e**), Ir (**f**).

Supplementary Table S5. PC score values for saliva and buffer samples.

	PC 1	PC 2
PBS	-0.97153<br>-0.97385	0.19364<br>-0.18509
Saliva collected before lunch (sample 1)	0.79139<br>0.82371	0.58968<br>0.54882
Saliva collected after lunch (sample 2)	0.86219<br>0.79735	-0.50276<br>-0.59871

Supplementary Table S6. PC score values for SARS-CoV-2-negative (samples 4, 6-11) and SARS-CoV-2-positive (samples 3 and 4) saliva samples.

Sample No.	PC 1	PC 2	PC 3	PC 4
4	-0.913<br>-0.918	0.040<br>-0.080	0.024<br>0.015	0.217<br>0.227
6	0.196<br>0.236	0.787<br>0.577	-0.321<br>-0.579	-0.152<br>-0.055
7	-0.395<br>-0.339	0.824<br>0.746	-0.206<br>-0.337	0.068<br>0.091
8	0.381<br>0.543	0.347<br>0.178	0.732<br>0.691	-0.258<br>-0.188
9	0.732<br>0.633	-0.073<br>-0.314	0.029<br>-0.046	0.560<br>0.582
10	0.849<br>0.777	0.092<br>-0.163	0.048<br>-0.115	0.405<br>0.521
11	-0.656<br>-0.514	-0.095<br>-0.102	0.545<br>0.729	0.174<br>0.072
3	0.559<br>0.571	-0.035<br>-0.292	-0.069<br>-0.147	-0.740<br>-0.591
5	-0.253<br>-0.248	-0.762<br>-0.808	-0.418<br>-0.450	-0.205<br>-0.124
